# Development and Validation of a Tool to Measure Gender Equality Among Adults in a Slum of Kolkata, India

**DOI:** 10.7759/cureus.86418

**Published:** 2025-06-20

**Authors:** Jyotika Singh, Bobby Paul, Rumelika Kumar, Monalisha Sahu, Rivu Basu

**Affiliations:** 1 Preventive and Social Medicine, All India Institute of Hygiene and Public Health, Kolkata, IND; 2 Occupational Health, All India Institute of Hygiene and Public Health, Kolkata, IND

**Keywords:** community-based tool, exploratory factor analysis, gender disparity, gender equality, psychometrics, questionnaire development and validation, scale development, social determinants of health, urban health

## Abstract

Introduction: Gender equality is a critical social determinant of health and well-being that influences an individual throughout the span of life. The conditions and constructs related to gender are highly specific and depend heavily on the social contexts in which individuals live. Therefore, the purpose of the present study was to develop and validate a tool to assess gender equality in the Indian context, particularly in a slum of Kolkata, where layered gender-based inequities exist.

Methods: The study was undertaken over the period of 12 months, from March 2024 to March 2025. A robust literature review and qualitative methods, such as in-depth interviews with experts, were used to develop the initial pool of items - 98 items that were refined and reduced to 23 after three rounds of Delphi among five experts and member checking. The questionnaire, containing the 23 items and sociodemographic details, was administered to a sample of 134 women and 96 men (a total of 230 individuals) residing in a slum of Kolkata. Statistical analyses were done in Statistical Product and Service Solutions (SPSS, version 16; IBM SPSS Statistics for Windows, Armonk, NY) and Jamovi v.2.6.13.

Results: The initial 23-item gender equality scale, developed through literature review and expert input, was administered to 230 adults in a Kolkata slum. Content validity led to the removal of five items with an item-content validity index (I-CVI) of < 0.8. Reliability analysis showed strong internal consistency (Cronbach’s α = 0.93), and one item was dropped for low item-total correlation. Exploratory factor analysis supported a three-factor solution consistent with the theoretical framework: access to resources, recognition and dignity, and participation in decision-making. All domains demonstrated strong internal consistency (α > 0.80) and moderate inter-domain correlations (r = 0.33-0.55), supporting their distinct yet related nature. The final scale comprised 21 items and showed good psychometric properties for use in similar community settings.

Conclusion: A reliable, context-specific tool to measure gender equality at the community level was developed and validated in a slum population of Kolkata. The scale demonstrated strong internal consistency and aligned with key conceptual domains. It holds potential for generating granular data to inform focused interventions targeting the gendered determinants of health.

## Introduction

The UN Women defines gender equality as “the equal rights, responsibilities and opportunities of women and men and girls and boys. Equality does not mean that women and men will become the same but that women’s and men’s rights, responsibilities, and opportunities will not depend on whether they are born male or female” [1]. Prevailing societal order places women, transgender, and non-binary individuals at a significant disadvantage in terms of access to education, livelihood, healthcare, social support, and power [2]. The Gender Gap Index 2024 reveals that only 68.1% of the gender gap has been closed worldwide, with women holding merely 26.1% of parliamentary seats and facing a persistent 20% wage gap [3].

There are multiple frameworks in use for the assessment of gender equality, and notable few are the ones used by the Convention on the Elimination of All Forms of Discrimination Against Women (CEDAW), Organisation for Economic Co-operation and Development (OECD)'s Social Institutions and Gender Index (SIGI), and World Economic Forum’s Global Gender Gap Index [3]. The CEDAW framework is built on the pillars of elimination of discrimination, substantive equality, and transforming stereotypes [4], while the OECD’s SIGI framework comprises the domains of discrimination in the family, restricted physical integrity, restricted access to resources, and restricted civil liberties [5]. The significance of these domains in gender equality is evident, although their influence on health is often exerted in a more indirect manner. From the perspective of health and well-being, gender remains a crucial determinant, and the present study aimed to construct a quantitative tool that can be used to gather granular data on the individual’s experience of gender. Therefore, the framework of gender equality given by the UN Women [1] was found to be the most suitable.

The UN Women has conceptualized the achievement of gender equality based on the following domains [1].

Equal access to resources: This includes livelihoods, recognition, reduction, and redistribution of unpaid care work, education, health, and other social services and public goods and exerts a direct influence on health and contributes significantly to the realization of an individual's highest potential [6].

Equal recognition and dignity: This includes challenging stereotypes related to traditional gender identities and roles, protection from gender-based violence, and assurance of bodily autonomy - stereotypes and rigid norms around gender identities and roles impact an individual’s mental well-being, gender-based violence is intimately associated with poor health outcomes, and bodily autonomy is imperative for living a life with dignity [7,8].

Equal participation: In multiple levels of decision-making, including participation in and leadership of collective action - the ability and opportunity to make a choice in various spaces, ranging from personal, interpersonal and at the community level is what enables individuals to shape their environment, and the conditions of their lives in a way that favors their well-being [9].

At present, there are multiple indices that measure the state of gender equality in defined geographical regions. These indices are essential to quantify and monitor the progress of countries, but they are, by nature, macroscopic and utilize proxies and population rates for multiple domains of gender equality. On preliminary review of the literature, a need was identified for a scale that could be administered in community-based settings where the individual was the subject of study and quantifiable, locally disaggregated data could be generated. Data generated by the use of the present tool have the potential to be used in designing interventions and policies that can directly address the gendered determinants of health. The tool can be used for the identification of gaps in specific domains, and targeted interventions may then be designed, which are appropriate and tailor-made to the community.

Objectives

The following are the objectives of this study: (1) to develop a quantitative tool to measure gender equality in a slum of Kolkata, India; (2) to evaluate the psychometric properties of the tool, including content validity and internal consistency; and (3) to determine the factor structure of the developed tool and examine internal consistency across subgroups.

## Materials and methods

The study was a cross-sectional tool validation study employing an exploratory sequential mixed methods approach. The qualitative strand informed the generation and refinement of items, while the quantitative strand involved administration of the tool and statistical analyses for establishing reliability and construct validity (QUAL --> QUAN). The scale development process was guided by published recommendations [10,11] and was conducted in the following steps: (1) formative review of literature and qualitative research for generation and refinement of item pool, (2) administration of scale to study participants, (3) psychometric analysis, and (4) scale validation.

Review of literature and qualitative research

A robust literature search was conducted on PubMed, Google Scholar, Medline, and JStor, and an item pool of 98 items was generated, corresponding to the subdomains within the three domains of gender equality as proposed by the UN Women. In-depth interviews (IDIs) were conducted with experts for the subdomains for which items could not be generated using the literature review. An IDI for the subdomain "education" was conducted with an educator working in the field for the past seven years. For the subdomain "access to social services and public goods", a gender studies expert was consulted, with experience in the field for five years. For the subdomain on "livelihoods", an IDI was conducted with an expert in social work working for labor rights for the past 10 years.

The Delphi process involved three iterative rounds of structured discussions with a panel of three experts from the fields of public health, health promotion, and gender studies. In each round, the experts were provided with the item pool and asked to rate each item on a 4-point Likert scale for relevance, clarity, and comprehensibility. Items that received an average rating below 2 on any of the three criteria were either modified or removed.

Consensus was defined a priori as ≥ 60% agreement among panelists on all three parameters. In each subsequent round, only items that failed to meet the consensus threshold were re-evaluated after modification based on expert feedback. The process concluded when full consensus was achieved on the final set of 28 items. Credibility of the process was established by member checking by two independent researchers, one who identified as a woman and one who identified as a man, and then the set of 28 items was taken forward for content validity.

These 28 items were subjected to content validity assessment by five experts from the fields of public health (two experts with a cumulative experience of 12 years in the field), health promotion (one expert with 13 years’ experience in the field), media and gender studies (one expert with five years’ experience working in the field), and sociology (one expert with 10 years’ experience). Items with a content validity index (CVI) less than 0.7 were removed. Thus, the final tool consisted of 23 items across three domains. The responses for all the items were on a 4-point Likert scale: Strongly agree (coded 1) to Strongly disagree (coded 4). Items 8, 9, 10, 12, 13, 14, 15, 20, 21, 22, and 23 were reverse-coded.

Administration of the Scale to Study Participants

The electoral roll of the study area was obtained, and a sampling frame was constructed. Adults aged 18-59 years who had been residing in the study area for one year or more were included. A total of 230 participants were selected by simple random sampling, and the scale, consisting of 23 items along with basic sociodemographic details, was administered.

Tool validation

Study Setting and Sample Size

The study was conducted in Chetla, a densely populated Kolkata slum, in the field practice area under the Urban Health Unit and Training Centre of the All India Institute of Hygiene and Public Health. Data were collected over a period of three months from June 2024 to August 2024 using a simple random sampling method. The scale was administered to 230 adults as the sample size was item dependent in a 1:10 ratio of items to respondents [12]. The data collected were used to perform reliability analysis and exploratory factor analysis (EFA).

Statistical Analysis

For the calculation of item-level CVI (I-CVI) and scale-level CVI based on the average method (S-CVI/ Ave), the scores for relevance were tabulated, and the percent agreement for each item (variable) was calculated. Items with CVI <0.8 were discarded, and the mean of I-CVI of remaining items was calculated for S-CVI/Ave [13]. A total of 23 variables were subjected to factor analysis to determine the underlying factor structure. Bartlett’s test of sphericity (with p value < 0.05) and Kaiser-Meyer-Olkin (KMO) measure of sampling adequacy (KMO value greater than 0.70) were used to assess the fitness of the variables for factor analysis [14]. Since EFA assumes that total variance can be partitioned into common and unique variance, it was found to be more suitable for the dataset [15]. The principal axis factoring method with oblique rotation was used as the factors (domains) were hypothesized to be intercorrelated [15].

Internal consistency of the scale was established using the following measures. The inter-item correlation matrix was examined, and the average inter-item correlation was assessed, with a desirable value between 0.15 and 0.50 [16]. For each item (variable), the item-total correlation was assessed, and a minimum item-total correlation of 0.20 was set to include items in the final scale [17]. Further, Cronbach’s alpha was calculated for the scale, and a value ≥0.70 was considered satisfactory [18]. All statistical analysis was performed in Statistical Product and Service Solutions (SPSS, version 16; IBM SPSS Statistics for Windows, Armonk, NY) and Jamovi v.2.6.13 (https://www.jamovi.org/).

## Results

Sample characteristics

Among the study participants, 58.3% were women and 41.7% were men, with the median age of 35.5 years (IQR (28, 46)). Moreover, 75.2% of the participants were Hindu by religion, while the rest were Muslims; 16.5% of the study participants were scheduled castes/scheduled tribes, castes that have been historically marginalized; and 19.1% belonged to other backward classes. The majority of the women were homemakers (61.9%), the second most common occupation among them being that of domestic work (14.1%). A third of the men (37.5%) were drivers, followed by 29.2% who were engaged in sales.

Content validity

The range of I-CVI was from 0.2 to 1; five items with I-CVI < 0.8 were identified and subsequently discarded; and S-CVI/Ave was 0.94, which was acceptable.

Reliability

Inter-item correlations averaged 0.30 and ranged from -0.71 to 0.79. Corrected item-total correlations for all items were > 0.20, except item 17 (-0.68). Cronbach’s alpha for the scale was 0.908, which was acceptable. Furthermore, Cronbach’s alpha of the scale increased to 0.93 upon removal of item 17. Hence, item 17 was dropped, and further analysis was conducted on the scale containing 22 variables (items). Reliability was calculated for each item as item-rest correlation, and reliability of the respective domains was calculated (as described in Table 1), along with item descriptive statistics. In addition, the reliability of domains and the scale overall were estimated separately among men and women (as shown in Table 2).

**Table 1:** Item descriptive statistics, item reliability, and domain reliability

Items	Item–rest correlation	Mean (SD)	Domain	Cronbach’s alpha of the domain
It is my sole responsibility to tend to household chores	0.791	2.23 (1.23)	Domain 1 – Access to Resources	0.897
Being a man/woman has made it tougher for me to find paid work	0.672	2.73 (1.12)		
At times, I am ill, and I still have to do household chores despite the illness	0.784	2.23 (1.31)		
Societal expectations make it difficult for me to pursue a higher education, even if I wish to	0.532	2.45 (1.04)		
I am concerned about going out late at night, because I am a man/woman	0.702	2.71 (1.06)		
I have had to discontinue my education against my wishes because of my gender	0.616	2.64 (1.13)		
Being a man/woman subjects me to unfair treatment at work	0.604	3.05 (0.917)		
I am expected to be the primary caregiver of children because of my gender	0.714	2.18 (1.32)		
I only get to eat after everyone else has finished eating, because of my gender	0.534	2.99 (1.07)		
I am able to operate a separate bank account	0.470	3.43 (1.08)		
A man/woman (opposite to the gender of the respondent) close to me uses or has used physical force against me	0.761	3.17 (1.05)	Domain 2 – Recognition and Dignity	0.868
I have been humiliated in front of others by a man/woman (opposite to the gender of the respondent) close to me	0.780	3.93 (1.03)		
I have the power to control when I have children	0.655	2.84 (1.06)		
I can have adequate portions of food, as much as I need	0.622	3.39 (0.843)		
I can say no to my partner when I do not wish to have sexual intercourse	0.613	2.96 (1.02)		
I have a say in decisions about my health	0.581	3.20 (0.815)		
I am able to lend/spend money as per personal need and interest	0.687	2.80 (0.848)	Domain 3 – Participation in Decision-Making	0.810
I feel confident enough to go for official formal work like the municipal corporation office, the court, or the police station	0.606	3.00 (0.89)		
I have a say regarding important decisions in the household	0.627	3.13 (1.04)		
I am able to save money for future personal use	0.445	2.27 (0.974)		
I feel I can voice out my concerns and opinions regarding things that affect my community	0.646	2.78 (1.02)		

**Table 2:** Internal consistency of domains and scale for men and women

Domain/Scale	Cronbach’s α (Men)	Cronbach’s α (Women)
Domain 1 – Access to Resources	0.912	0.881
Domain 2 – Recognition and Dignity	0.885	0.856
Domain 3 – Participation in decision - making	0.822	0.802
Scale Overall	0.943	0.913

Exploratory factor analysis

**Figure 1:**
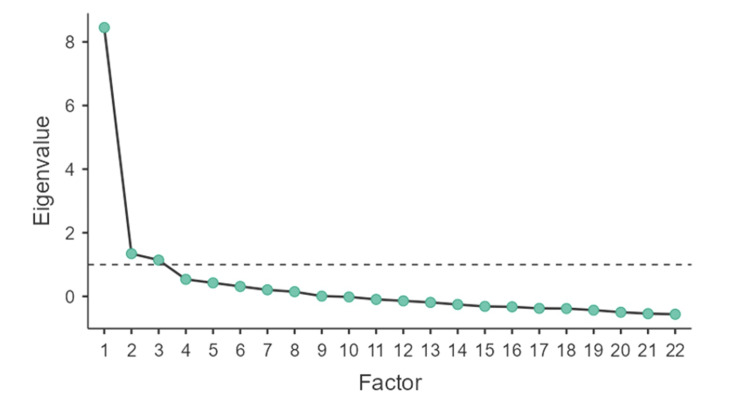
Screen plot showing the distribution of eigenvalues against the factors

EFA using 22 variables (Bartlett’s test of sphericity, p value < 0.001, and KMO = 0.82) was run using the principal axis factoring method in combination with oblimin rotation, and a three-factor solution was obtained, as shown in the scree plot (Figure 1).

The three-factor solution was consistent with the theoretical framework of gender equality [1]; therefore, the factors were considered as the domains of gender equality. Factor loadings are shown in Table 3. All but one item (variable) had factor loadings > 0.40. The item (health issue prioritization) demonstrated low factor loadings and loaded equally on two distinct factors and was therefore dropped from the final scale.

**Table 3:** Factor loadings after exploratory factor analysis with oblique rotation Note: The "principal axis factoring” extraction method was used in combination with “oblimin” rotation.

Items	Factor 1	Factor 2	Factor 3
It is my sole responsibility to tend to household chores	0.775	-	-
Being a man/ woman has made it tougher for me to find paid work	0.747	-	-
At times, I am ill, I still have to do household chores despite the illness	0.734	-	-
Societal expectations make it difficult for me to pursue a higher education, even if I wish to	0.706	-	-
I am concerned about going out late at night, because I am a man/ woman	0.701	-	-
I have had to discontinue my education against my wishes because of my gender	0.700	-	-
Being a man/ woman subjects me to unfair treatment at work	0.534	-	-
I am expected to be the primary caregiver of children because of my gender	0.521	-	-
I only get to eat after everyone else has finished eating, because of my gender	0.480	-	-
I am able to operate a separate bank account	0.412	-	-
A man/ woman (opposite to gender of respondent) close to me uses or has used physical force against me	-	0.889	-
I have been humiliated in front of others by a man/ woman (opposite to gender of respondent) close to me	-	0.847	-
I have the power to control when I have children	-	0.605	-
I can have adequate portions of food, as much as I need	-	0.585	-
I can say no to my partner when I do not wish to have sexual intercourse	-	0.524	-
I have a say in decisions about my health	-	0.425	-
My health issues are prioritised among others in my family	-	0.396	0.389
I am able to lend/ spend money as per personal need and interest	-	-	0.849
I feel confident enough to go for official formal work like the municipal corporation office, the court or the police station	-	-	0.653
I have a say regarding important decisions in the household	-	-	0.630
I am able to save money for future personal use	-	-	0.571
I feel I can voice out my concerns and opinions regarding things that affect my community	-	-	0.554

Inter-domain correlations among the three domains were also found and are shown in Table 4. Domain correlations ranged from 0.332 to 0.551, showing that the domains are moderately correlated, justifying the choice for an oblique rotation.

**Table 4:** Spearman's correlation matrix of tool domains: multidimensionality of domains

Items	Domain 1	Domain 2	Domain 3
Domain 1	1	0.413	0.551
Domain 2	0.413	1	0.332
Domain 3	0.551	0.332	1

On the basis of the content of items that loaded on the domains, each domain was given nomenclature consistent with that in the theoretical framework [1]. Hence, Domain 1 was access to resources, Domain 2 was recognition and dignity, and Domain 3 was participation in decision-making.

## Discussion

The present study aimed to develop and validate a quantitative tool to assess gender equality in community-based settings among adults in a slum of Kolkata. The three-factor solution that emerged - access to resources, recognition and dignity, and participation in decision-making consistent with the conceptual domains proposed by the UN Women and also aligns with findings from similar settings.

The internal consistency of the scale (Cronbach’s α = 0.93) affirms its reliability. Although no tools exist to measure the same construct, tools measuring related constructs have been considered for the purpose of this discussion. While assessing psychometrics of the Malaysian version of the Gender Equitable Men (GEM) Scale, Rashid et al. [19] reported Cronbach’s α with a range from 0.778 to 0.921, which is similar to the present study. Filippo et al. [20] reported the internal consistency of the adapted Gender Relations Scale (aGRS) (Cronbach’s α = 0.75) among young women in South Africa as part of a randomized controlled trial.

The domain “access to resources” captured gender-based differentials in access to education, employment, and the division of unpaid work - factors that have been extensively reported as key to structural gender inequality in communities [6,21,22]. The item “I only get to eat after everyone else has finished eating, because of my gender”, which was loaded onto this domain, is indicative of the pervasive gender biases even in basic needs of survival, which is congruent to what was reported in the systematic review by Harris-Fry et al. [23]. The item “I am concerned about going out late at night, because I am a man/ woman” captures gendered patterns of exclusion from urban spaces and reiterates that the valuing of some social groups over others is maintained through the interactions and access to differentiated spaces of the city, as has been described by Beebeejaun [24].

The second domain, “recognition and dignity”, clustered items related to experiences of gender-based violence, autonomy over one’s body with reference to health and reproductive choices. Items such as “I can say no to my partner when I do not wish to have sexual intercourse” and “I have a say in decisions about my health” reflect that bodily autonomy is pertinent to the enhancement of power, choice, and dignity among individuals who have previously been denied these [8].

“Participation in decision-making”, the third domain, reflects the respondent’s influence in household and community-level decisions. The significant loadings on items related to financial decision-making and civic engagement demonstrate that gender equality extends beyond personal circumstances into collective life. A cluster randomised controlled trial in Nigeria by OlaOlorun et al. established financial decision-making and household decision-making aspects to be key determinants to achieving gender equitable conditions [25].

The moderate correlations between the three domains (ranging from 0.332 to 0.551) validate the interdependent nature of these constructs. This interrelation has also been described in the theoretical framework of empowerment by Kabeer, who conceptualizes the empowerment of women as an interplay between resources, agency, and achievement [26]. In the setting of rural West Bengal, Chakraborty et al. [27] have constructed an index of women empowerment consisting of the domains of decision-making power, attitudes to gender based violence, and social independence, to which the present study is congruent.

Thus, the developed tool shows strong psychometric properties and contextual relevance. It holds potential for community-level assessment and tracking of gender equality, especially in urban poor settings where granular, individual-level data are lacking.

Strengths

The study has some notable strengths; for example, the tool is conceptually anchored in the globally recognized UN Women framework, ensuring theoretical robustness while being adapted for local relevance through expert input and community validation. An exploratory sequential mixed methods design further strengthens the study, with qualitative methods informing item generation, followed by rigorous quantitative validation in the form of content validity and internal consistency measurement. Gender equality is an abstract construct, and the present study is a step in a novel direction aimed at the quantification of this construct, which is so pervasive and intricately linked with human health and well-being.

Limitations

The developed tool suffers from the limitation of failing to account for genders beyond the binary. The tool could be modified for use with non-binary persons by modifying items with gendered terminology and addition of items that capture the non-binary experience of gender. In future iterations of the tool, we intend to incorporate the lived experiences of non-binary and transgender individuals through extensive qualitative interviews and community consultation, ensuring that their perspectives meaningfully inform item development and content validity. Further, item analysis using item response theory could be done for the refinement of individual items. The study was conducted in a single urban slum in Kolkata, which may limit the generalizability of the tool to other geographic or sociocultural settings. A formal pilot study would have enhanced the semantic validity of the tool but could not be undertaken due to resource constraints. Gender norms and lived experiences can vary significantly across regions, cultures, and socioeconomic groups. Therefore, further validation in diverse populations is needed before broader application. Confirmation of factor structure and scoring needs to be developed for application into community-level surveys and enhanced generalizability. Potential biases include social desirability bias, owing to the self-reported nature of responses to items related to gender roles, autonomy, and violence. Items may have been framed in an expert-driven manner in the initial qualitative strand; however, this was countered by member checking and content validity assessment.

## Conclusions

The present study was conducted to develop and validate a reliable and contextually grounded instrument for measuring gender equality at the individual level in a marginalized urban population. The study employed a mixed methods approach to capture culturally appropriate and grounded information, which is applicable to the slum setting in Kolkata. The tool demonstrated robust internal consistency and aligned with established conceptual domains of gender equality. Its application in community-based research can aid in capturing nuanced, locally relevant data to inform targeted gender equity initiatives. Further psychometric evaluation, including confirmatory factor analysis and item response theory, is planned to define scoring and enhance the tool’s generalizability.
